# Integrated Care Model *Salut+Social* Assessment by Professionals, Informal Caregivers and Chronic or Social Dependent Patients: A Qualitative Study

**DOI:** 10.3390/ijerph192315467

**Published:** 2022-11-22

**Authors:** Ester Gavaldà-Espelta, Maria del Mar Lleixà-Fortuño, Carina Aguilar Martín, Macarena Pozo, Maria Ferré-Ferraté, Begoña Tomàs-Navarro, Claudia Curto-Romeu, Jorgina Lucas-Noll, Jordi Baucells-Lluis, Alessandra Queiroga Gonçalves, Carmen Ferré-Grau

**Affiliations:** 1 Direcció d’Atenció Primària Terres de l’Ebre, Gerència Territorial Terres de l’Ebre, Institut Català de la Salut, 43500 Tortosa, Spain; 2 Departament d’Infermeria, Programa de Doctorat Infermeria i Salut, Universitat Rovira i Virgili, 43002 Tarragona, Spain; 3 Departament d’Igualtat i Feminismes a les Terres de l’Ebre, Direcció de Serveis Territorials a les Terres de l’Ebre, Generalitat de Catalunya, 43500 Tortosa, Spain; 4 Unitat d’Avaluació, Direcció d’Atenció Primària Terres de l’Ebre, Institut Català de la Salut, 43500 Tortosa, Spain; 5 Unitat de Suport a la Recerca Terres de l’Ebre, Fundació Institut Universitari per a la Recerca a l’Atenció Primària de Salut Jordi Gol i Gurina (IDIAPJGol), 43500 Tortosa, Spain; 6 Gerència Territorial Terres de l’Ebre, Institut Català de la Salut, 43500 Tortosa, Spain; 7 Equip d’Atenció Primària Amposta, Gerència Territorial Terres de l’Ebre, Institut Català de la Salut, 43870 Amposta, Spain; 8 Direcció de Sistemes d’Informació i Comunicació, Gerència Territorial Terres de l’Ebre, Institut Català de la Salut, 43500 Tortosa, Spain; 9 Unitat Docent de Medicina de Familia i Comunitària, Tortosa-Terres de l’Ebre, Institut Català de la Salut, 43500 Tortosa, Spain

**Keywords:** chronic disease, primary health care, social work, qualitative research, focus group, interview

## Abstract

We explored the views of the professionals (from primary care and social services) and users (caregivers and patients) who participated in the clinical trial of the Salut+Social integrated care model to identify the implementation barriers and facilitators, to assess the impact on health and wellbeing and to obtain an assessment of the program. A qualitative descriptive study with a pragmatic, utilitarian approach was performed. Participants were recruited by purposive and convenience sampling. A focus group (FG) and in-depth interviews were conducted with professionals and users, respectively. Thematic content analysis was employed. A total of 11 professionals and 8 users participated in the FG and interviews, respectively. Seven themes were identified: (1) contextualizing the previous scenario; (2) achievements of the program from the professionals’ perspective; (3) facilitators and barriers of the integrated care model; (4) proposals for improving the integrated care model; (5) users’ assessment of the care received within the program framework; (6) users’ perception of the impact on health and wellbeing; (7) users’ demands for better care. Professionals reported improved coordination between services and highlighted the need for a protocol for emergencies and to strengthen community orientation. Users proposed more frequent home visits. This study shows the acceptability of the new model by professionals and the users’ satisfaction with the care received.

## 1. Introduction

The epidemiological transition in recent decades towards an aging population, leading to an increase in morbidity, is one of the most important sociological changes of the 21st century in developed countries [1]. Multimorbidity and chronicity entail new health and social needs for an aged population due to the impact on their quality of life and functional status. The major challenge for the care systems is to satisfy the complex needs of these patients, simultaneously heightening the importance of their rational and cost-effective use [2,3]. Providing caregivers with means to lessen their burden is another challenge that has arisen [4]. According to the Catalan Health Survey of 2018, chronic health problems become more prevalent with age and are more common in disadvantaged social classes, in women and in those with a lower level of educational attainment [5]. In addition to these conditioning factors, the complexity of care is usually related to several dimensions, including the difficulties of managing the response to the health needs of patients, the social resources available to them (family, caregiver, community services) and the caring environment (professionals, caring system) [6].

To adapt to the aforementioned recent demographic and epidemiological changes, the previous Catalan Health Plan (2016–2020) promoted the creation of bottom-up projects promoting organizationally and technologically integrated care [7]; this is the context in which the present study was developed. More specifically, Catalan laws established different territorial divisions for social and health services, which has led to a health system with centralized governance, but social services that are decentralized and with important local competencies [8]. Innovative caring models, therefore, require territorial governance environments, integrated care adapted to the specific needs of the current population, and professionals and citizens who are involved in the design of more efficient, patient-centered care models [9]. This means moving from a reactive approach that is focused on individual diseases and is fragmented and paternalistic to more proactive, integrated and empowering models that encourage the active participation of professionals and communities in training, self-management and decision-making [10]. 

A new integrated care model (the “Salut+Social model”) was designed in Catalonia’s primary care (PC) context with the aim of promoting the coordination of health and social services to improve the care of chronic and socially dependent patients and to reduce the burden on informal caregivers. The previous model was characterized by the uncoordinated work between sectors with a lack of a holistic approach in which each service addressed only immediate health and social needs. It was also characterized by excessive visits of the patients to service locations and duplication of some tasks carried out by professionals of both sectors, such as preventive evaluation of social risk, cognitive impairment and dependency grade. In this model, delays occurred in obtaining social assistance since patients began to seek social services only in the very advanced stages of the disease due to the need for family support. The new model aimed to promote person-centered care with a joint approach of both sectors since disease diagnosis to anticipate and take adequate actions to provide for patients’ necessities. It promoted joint visits with professionals of health and social services, especially domiciliary, and the assignment of professional roles to reduce the duplication of professionals’ tasks. In practice, during monthly meetings of the work team, common cases of social and health services were addressed and discussed, with the aim of establishing a joint individualized intervention plan [11]. Moreover, a new Information and Communication Technology (ICT) tool (web-based and a mobile app) was created based on data from the electronic clinical history of the Catalan Institute of Health (CIH) and social data from the workstations of social services, which allowed data integration and registration of the planned actions. 

The Salut+Social model was conceived within the Medical Research Council (MRC) framework, which encourages developers and evaluators of interventions to understand how the intervention causes change, the mechanisms of impact, and the barriers to and facilitators of implementation in order to evaluate its effectiveness in everyday practice [12]. We designed two phases of evaluations with distinct methodologies: Phase 1: testing of the model in daily practice through a pilot study with a quantitative evaluation of its effectiveness; Phase 2: a qualitative assessment of users’ experiences and views with focus groups and interviews. With the results of both phases, the research team aims to: 1. re-design the model, taking into account the encountered barriers and facilitators for the implementation; 2. implement and evaluate the re-designed model in more areas of Catalonia with a randomized clinical trial.

The effectiveness of the model was evaluated through a clinical trial and was characterized by professionals’ training in the use of the app and the knowledge of service portfolios and the assignment of new professional roles based on a multidisciplinary approach. Patients and caregivers received a personalized assessment with follow-up to evaluate quality of life, treatment adherence, medical service use and caregiver burden. More detailed information is available in the published study protocol [13]. Results from the clinical trial indicate that this strategy could be beneficial to patients’ health-related quality of life, treatment adherence, caregiver burden and access to, and granting of help from, social services (data under review). 

Qualitative research often focuses on participants’ experience with the aim of describing and explaining that experience and, in this case, how they receive and personally perceive the intervention [14]. In this study, we explore the views of the professionals and users (caregivers and patients) who participated in the clinical trial of the Salut+Social integrated care model in order to identify the implementation barriers and facilitators, to assess the impact on health and wellbeing (users’ views) and to obtain an assessment of the program. The information obtained must identify the components needing improvement that may help to model the basis of integrated health and social care in Catalonia.

## 2. Materials and Methods

### 2.1. Research Design

Taking a pragmatic, utilitarian approach, we conducted a qualitative descriptive study [15]. This approach is suitable for evaluating programs, as it is based on the adoption of standards that require evaluations to be useful, precise, ethical and practical rather than on the pursuit of more profound, theoretical models [15]. The study follows the Standards for Reporting Qualitative Research (SRQR) (Supplementary File S1).

A focus group (FG) and in-depth interviews were conducted with professionals and users (caregivers and patients), respectively, who had participated in the intervention. The FG was held to understand professionals’ experiences and opinions about the new model while offering a space to generate discussion among participants to compare their responses in a small group [16]. The research team developed a topic-scheduled guide under the following headings: acquisition of new information/knowledge from the reciprocal service (health or social) since participation in the program; advantages and disadvantages of the new integrated care model; usefulness of the Salut+Social app in daily practice; areas for the improvement of the coordination between the two services. 

To foster an ambience in which the users could feel confident, interviews were conducted to explore personal experiences and the direct benefits obtained from implementing the program, as well as opinions about how to improve the program. The following headings were considered in the guide to the scheduled interview topics: experiences within the program, opinions about the care provided by health and social services professionals, and the impact of the care received inside the program on the daily life and health of caregivers and patients. The professionals’ FG was carried out in a training room at a Primary Care Center (PCC); users were interviewed at the PCC or in their homes. 

### 2.2. Participants, Sampling and Recruitment

The participants in the study included professionals (nurses, general practitioners, and social workers) and users (caregivers and patients) who had previously participated in the intervention that was implemented at two primary care centers (PCCs) of the Institut Català de la Salut (ICS) (Ribera d’Ebre, Gerència Territorial Terres de l’Ebre, Catalunya). The professionals’ FG was carried out in a training room of one of the PCCs, and users’ interviews were held either at the PCC or at their homes. 

All the professionals who had participated in the intervention were invited to join the FG by an e-mail sent by the Principal Investigator. We intended that those who replied to the e-mail and expressed an interest in taking part would be purposively sampled [17] to ensure the maximum discourse diversity of professionals with respect to their age, gender, professional category (general practitioner, nurse, social worker) and number of years in that employment. Nevertheless, because the number of interested professionals was below 12 (the maximum permitted for a focus group), they were all included, making the final sample also one of convenience. For the interviews, patients’ age, gender and health or social condition were considered to ensure the heterogeneity of the sample. A list of patients in the study was drawn up, and their referral case manager contacted their caregivers by phone, inviting them to participate. A joint decision by the research team was made about selecting the most suitable person to interview (the patient or their caregiver) and the place of interview (PCC or home). A reminder was sent 24 h before the scheduled FG or interview.

### 2.3. Data Collection and Analysis

The FG was held after the intervention period of the study (November 2019), and the interviews were conducted up to one year after the intervention had finished (November 2019–November 2020). Data were recorded by digital audio. The FG was conducted by an experienced moderator (a nurse with a Ph.D. and qualitative research expertise) and an observer (an experienced nurse); interviews were performed by an experienced moderator with a biomedical Ph.D. Participants and members of the research team had had no contact before the sessions. 

The FG and interviews lasted 60 min 33 s and a mean of 19 min 24 s, respectively. The FG and interviews were transcribed verbatim and anonymized by an external interviewer to guarantee confidentiality. Field notes were written during and after sessions. Flexible and iterative thematic content analysis was carried out. It consisted of six phases: familiarization with the data, generalization of initial codes, a search for themes, a review of the themes, the definition of the final themes and write-up [18,19]. Concretely, familiarization with the data was conducted by reading the material and registration of preliminary analytical intuitions. For the generalization of initial codes and searching of themes, the text corpus of the focus group and of the first two interviews was analyzed by A.Q.G. to identify codes and categorize the most relevant units of meaning. Consecutively, a triangulation was performed with two other members of the research team (M.P and E.G.E) to obtain a consensus on the most important emerging themes. The remaining interviews were analyzed considering the results obtained with the first two interviews, being cross-analyzed, and compared. Subsequently, a second triangulation with the same research team was performed for the revision and discussion of the final themes and to reach a consensus. Interviews were discontinued when no new theme emerged from the data.

Reflexivity was carried out in the different study phases with the aim of validating the data. The research team triangulated the coding and the final categories, determined by consensus following discussion among the research team, thereby ensuring that different perspectives were represented [19]. 

## 3. Results

### 3.1. Description of Participants

Of the 28 professionals (13 nurses, 8 social workers and 7 GPs) involved in the intervention, 11 participated in the FG (9 females, mean age: 46.8 ± 6.7 years). The sample of users comprised 7 caregivers (5 females, mean age: 58.4 ± 10.3 years) and 1 female patient. The characteristics of the participants are described in Table 1 and Table 2. 

### 3.2. Themes

Seven key themes were identified from the FG and interviews. The first four themes covered professionals’ opinions about the care provided before the introduction of the new integrated care model, the achievements after the introduction of the program, and the facilitators of and barriers to program implementation and proposals for improving the integrated care model. Themes five to seven were related to users’ opinions about the care received within the framework of the program, its impact on their health and wellbeing and users’ demands for better care.

### 3.3. Professionals’ Opinions

#### 3.3.1. Theme 1: Contextualizing the Previous Scenario

Professionals contextualized the way they previously worked and highlighted difficulties in communication and the coordination of their actions between the social and health sectors. Ignorance of the service portfolio of each other’s sector and the lack of shared, systematically compiled records were cited as being the cause of discoordination, which made it difficult to monitor patients adequately.

*“To know their portfolio of services and that they are also familiar with our portfolio of services, right? I also think it is very important”* (general practitioner, P3).

They reported that communication between the sectors customarily took place over the phone, which slowed down the process. Additionally, professionals were used to working in a non-proactive way since they only intervened in social or health problems.

*“… when there is a problem, we act. Then they said “well, you have to act before there is a problem”, which is being proactive”* (nurse, P4).

Some professionals were of the opinion that, since the study area is rural, the dispersed distribution of the population adds to the difficulties of coordination and communication. However, there were some contrary views since, in some areas, the social and health services were located close to each other, favoring their coordination.

#### 3.3.2. Theme 2: Achievements of the Program from the Professionals’ Perspective

The new app was described by the professionals as being a highly useful and dynamic tool for managing cases in the context of the great need for communication between health, social and community services. They unanimously valued the new integrated healthcare model very positively since it helped them improve coordination between the health and social services.

*“... apart from the application, it has also served us for this, to coordinate ourselves much better”* (social worker, P9).

Furthermore, all the professionals agreed that they acquired knowledge about the contributions of the various professional roles and services provided and reported that they perceived an improvement in the provision of services and patient care as benefits of the program.

*“…that I, for example, have met her, that I know what portfolio of services they have, also means that I am much more proactive with my service...... “come on, ask about the law of dependency”..., I know what I can offer users, I move them a little more, activate a little more... and I think they are already benefiting from this”* (nurse, P2).

#### 3.3.3. Theme 3: Facilitators of and Barriers to the Integrated Care Model

With regard to the facilitators, participants reported that the app helped improve the coordination between sectors since it allows for data registration and the unification of procedures that facilitated efficient follow-up and avoided duplication of tasks. As a result of these improvements, they expressed great satisfaction with the reduction in bureaucracy related to case management and unnecessary visits and with the more efficient provision of care.

*“So, yes... I think that it helped us. And to be more efficient, right? Maybe fewer visits, both for us and for you (social workers), has solved our problems”* (general practitioner, P6).

Likewise, the professionals pointed out that, within the framework of the program, common spaces and regular in-person multidisciplinary meetings were set up to share and discuss the cases they worked on together and that these meetings were crucial for boosting personal and professional relationships and for facilitating coordination. The professionals valued the possibility of knowing each other personally since this helped coordinate their actions. They also appreciated making joint home visits since this enabled them to make a broader diagnosis of situations and to provide a better response, which implies the better provision of patient care.

*“The Salut+Social program, what it has achieved is this, improving coordination, that we know each other, that we face each other, that we talk to each other, that we see each other every month or two, that we share information…”* (general practitioner, P7).

Some barriers were identified by the professionals. In the first place, they highlighted bureaucracy in granting processes from social services. They considered that service procedures took too long and that patients perceived the system failure and often preferred not to ask for help because they had no expectation of accessing any benefits.

*”... we have met quite a few people who had everything locked up in a drawer and who had not finished processing (social procedures)”* (general practitioner, P3).

Second, all the professionals stressed that an important drawback was the limited ability of the app specifically and the program overall to respond to emergencies. They complained that in the case of emergencies, they needed to go back to the old communication route and use the phone because the app was not suitable for alert notifications or real-time communication. During the implementation of the program, they became aware of the lack of protocols for social emergencies and hospital admission criteria and cited examples of distressing situations.

*“What happens is that when there is a serious emergency, which has to be dealt with quickly... Really, when you find yourself in that situation, it’s very overwhelming because there is no protocol in place, which I think is what needs to be worked on”* (general practitioner, P6).

Other salient factors identified by professionals as barriers were a shortage of personnel and excessive workloads. These culminated in an initial reluctance of some professionals to participate in the program. The high turnover of health professionals was also highlighted as a barrier.

*“Well, on a professional level, we all have quite a heavy workload, don’t we? You have a lot of visits... Then you think that this is extra work, don’t you? And you have to change the mentality of the person who, little by little, by using it, sees that it is not extra work, but that it is productive and that it can help them at specific moments…”* (general practitioner, P3).

#### 3.3.4. Theme 4: Proposals for Improving the Integrated Care Model

Professional participants were of the opinion that it is important to continue working on the coordination between the two sectors and that all professionals should incorporate the use of the program into their routines. They highlighted the need for the program to be community-oriented, with more home visits and more proactive actions, such as the early detection of indicators of vulnerability. Additionally, the existence of the new model should be emphasized to users since they usually confuse it with the level of attention normally provided and because this might be important for strengthening the program.

*“… we must do what we’ve talked about many times, to change the way we work... go into the community more, go out more, to the homes of chronic patients and do fewer things sometimes in the consultation... they don’t contribute much to the patient…”* (general practitioner, P3).

*“… above all, more proactive... because if it’s a person who lives alone, who is 88 years old … You process the degree (of dependency) and you can process anything, because you already have this person identified, and then everything is easier. Because... otherwise, if you have to start from scratch…”* (nurse, P5).

The following concrete actions were proposed by the professionals: to share physical spaces for a few hours a week and to retain the support of a case manager for the general coordination of patient-level integrated care and to coordinate with the hospital social services and the socio-sanitary services to respond to emergencies and for post-admission follow-up. 

Regarding this general concern about the program’s lack of immediacy and its limitations in dealing with emergencies, professionals suggested that these difficulties could be solved by improving the accessibility and incorporating a notification system in the app. They proposed that the app should be integrated into their usual database/browser to enable it to be directly accessed. Other proposals to improve the app and its use were to incorporate a direct communication (chat) function and to incorporate a meeting management section.

*“...the application itself is fine... that is, it has made it easier for us to share information and it has the difficulty that, for both the social and health services, we have to enter (the information) in a different part, which is outside our work’s usual computer program. This makes it difficult, but accepting that difficulty I think it is an important improvement”* (general practitioner, P3).

### 3.4. Users’ Opinions

#### 3.4.1. Theme 5: Users’ Assessment of the Care Received within the Framework of the Program

Users positively rated the coordinated work between social and health professionals, the information about the disposable resources and the care received as part of the program. They commented on the benefits of receiving a follow-up visit at home to ascertain the state of patients’ health and caregivers’ needs, which saves on travel, particularly benefiting patients with mobility difficulties.

*“The truth is that I am very satisfied, because all the resources, she (the social worker) has been there to tell us “This is what is set out in law and you can do it, that too…”* (P1).

*“... it is a problem to go from here to there. I already go enough (to the PC center) for the pills...”* (C4).

The program promoted the rationalization of procedures and access to resources, especially those for processing dependency and shared decision making.

*“… they (social workers) give you options, … they asked me… “Do you want her to go to the day center? It will be good for her, not being inside the house, locked up all the time…”* (C1).

Caregivers were of the opinion that the support of social services had given them a sense of security during the process of allocating resources, especially because many families did not have any kind of support to help them manage these procedures.

*“It also gives you more security in knowing that, if you have a problem, they will solve it for you”* (C1).

*“This program is ideal, because you (social workers) have to manage everything for him... there are many families who do not have anyone who can handle these things for them”* (C3).

Communication with professionals, especially social workers, was improved by the availability of a telephone line and WhatsApp, which the users considered to be effective. Although communication with health professionals also improved, one caregiver claimed to have had the usual difficulties with calling the health center to contact health professionals.

#### 3.4.2. Theme 6: Users’ Perceptions of the Impact on Health and Wellbeing

The users of the program did not notice any significant impact on their daily activities following the implementation of the program. However, all caregivers reported having received the help of a nurse assistant for a few hours a week, arranged by social services. They stated that this benefit gave them time for themselves, with some discourses suggesting a decrease in their sense of being overloaded. One caregiver, for example, expressed the feeling of calm they gained because they no longer had to leave their relative alone when they needed to go out.

*“… now I have an hour, before I had no one (nurse assistant). I used to leave her on the bed or sitting on the couch and went shopping. She was alone. Now I’m calmer about this...”* (C3).

Participation in the program also engendered emotional wellbeing because the users reported feeling good as a result of the professionals making home visits and showing an interest in them. This was highlighted as very positive, especially for elderly people, with a concrete example of mood improvement resulting from the visit of the nurse assistant.

*“…my mother also becomes happy, “They remember me, at least”. Older people value it... older people who are alone, that they come to see you, is a lot for them”* (C1).

#### 3.4.3. Theme 7: Users’ Demands for a Better Care

Basically, the users proposed not only increasing the frequency of routine visits for better patient control and caregiver support but also making health education about patient care procedures and the organization of the home environment to improve patient comfort.

*“... I don’t know if it they are thinking that they can do that “Every two months I will do a home visit” and that they value the patient, they value the caregiver, they value the environment”*(C2).

An Alzheimer’s patient’s caregiver requested additional follow-up after realizing his relative was rapidly losing his cognitive abilities.

*“...my father is like this and I want my father to be valued more, because every day he is losing more”* (C4).

## 4. Discussion

The present study is based on the qualitative assessment by health and social professionals of the new Salut+Social integrated care model. It suggests the improvement of coordination between the health and social sectors in a territory of south Catalonia and the acceptability of the model by professionals. It also assessed the opinions of PC users about the care received in the framework of the program, indicating satisfaction with the care provided.

Our study area is characterized by a growing aging population with chronic diseases and multimorbidity [13]. The current scientific literature suggests that integrated care is of greater importance for people with multimorbidity [20] due to the risk of fragmentation of care and the subsequent generation of poor outcomes in this population [21]. However, until now, there has been a lack of studies about the effectiveness of integrated health and social care programs and about their qualitative assessment [22,23]. We provide here what we believe to be the first qualitative analysis of professionals’ experiences and opinions of a new integrated care model in Spain for PC patients with chronic disease or social conditions that require social service care.

The professionals who participated in the intervention reported that before the implementation of the integrated care model, the health and social services did not work in a coordinated way, and there was a lack of knowledge about the reciprocal service portfolios in the two sectors. After implementing the program, the professionals knew each other personally and began to work in an interdisciplinary manner. This had a positive influence on the coordinated actions, which focused on taking a person-centered approach. Knowing other professionals personally and holding regular interdisciplinary meetings was identified as beneficial, as it facilitated communication and coordinated actions. Some studies have recognized the importance of interdisciplinary teams working together and of interprofessional communication in integrated care programs [24,25]. A qualitative study involving stakeholders from 17 European integrated care programs for patients with complex needs highlighted that the alignment of services and good relationships between professionals based on trust and facilitated by continuous communication are elements of a well-functioning integrated care process [26]. In a qualitative evaluation of an integrated care program in London, the mistrust among professionals observed at the beginning of the program seemed to change over time, giving rise to a more collaborative work culture [21]. Apart from the improvement experienced in the coordination of actions, the professionals reported that the creation of a new app with a systematic common registry allowed user care to be improved, with the optimization of visits, better follow-up of patients and a better offer of services in both sectors. 

On the other hand, the professionals reported that problems existed before the program was implemented. These were structural barriers, such as the lack of workforce and the excess workload and were a cause of the professionals’ reluctance. Distrust of the sustainability of the program was perceived, arising from the high turnover of PC health professionals. Some qualitative studies have discussed the need for time, resources and funding for planning integrated care programs [21,24], the lack of which constitutes an important barrier, for example, to providing access to formal dementia care [27] or high-quality integrated care for people living with dementia [28]. Our study included some patients with dementia, and their caregivers remarked upon the need for more frequent and continuous care because of the rapid progress of the disease. Staff shortages and insufficiently financed services were identified as barriers to access to and use of formal dementia care, which caused dissatisfaction among professionals [27].

As this is a project involving chronic patients who are of advanced age or in delicate health, the new integrated care model has prompted more joint actions between health professionals and social workers at the home level, which was identified by professionals and users as a very important benefit of the program. On the other hand, professionals raised their concern about the need for more community-level work with proactive actions, and all users requested more frequent home visits. 

The informal caregivers in the present project were patients’ relatives, and due to the patients’ characteristics, they participated actively in the follow-up and joint decision-making with the professionals within the program. In a European study of eight countries where the barriers and facilitators of access to and use of formal care for patients with dementia were evaluated, informal caregivers reported that they would like their experience and knowledge to be valued and felt that they should be considered as partners in care provision [27]. Another study indicated the importance of the role of the informal caregiver in shared decision-making in integrated care programs [24]. 

An important barrier to the program, which caused concern and anguish among professionals, was the limited ability of the program to be of value in emergency situations due to the lack of a protocol for joint action with the hospital social service and the socio-health service. The lack of procedures common to emergency departments and health and social care professionals is a frequent drawback worldwide [29]. Previous studies conducted by emergency departments have indicated the benefits of this kind of interdisciplinary work [30]. The increasing attendance of members of the older population in emergency departments is a challenging reality that requires the promotion of strategies to identify cost-effective models of care [31,32]. Although only limited evidence has been available to date, the Australian experience has shown a degree of effectiveness in improving patient outcomes [33].

The slowness of carrying out procedures in the social ambit was another limitation reported by professionals, which could, in their opinion, cause many patients to give up fighting to receive social service benefits. In some qualitative studies of dementia, informal caregivers have described their perception that access to services was confusing or that they were poorly organized [34,35], which could add to their stress [24]. However, in their interviews, the users of our study expressed their satisfaction since the program brought the social workers closer to the patients of the home care service, and there was even a telephone number available for rapid attention.

Caring for a family member could be a satisfying personal experience, but problems related to physical, psychological, and financial burdens requiring adequate support could also arise [4]. In Europe, 14.4% of the adults (52 million people) between 18 and 74 years of age, predominantly women, provide informal care to dependent family members on a regular basis per week. In Spain, between 25 and 30% of the women aged 45–64 years old provide informal care [36]. In this country, informal caregivers benefit from the Law for the Promotion of Personal Autonomy and Care for Dependent People (Law 39/2006), which foments actions such as training programs and measures to attend to periods of break [37]. As most of those interviewed in our study were informal caregivers of people receiving home care and with a certain degree of dependency, they identified the reduction in the care burden from receiving several hours’ work by a nurse assistant as being another benefit obtained through social services. Caregivers reported improved emotional wellbeing in the form of a sense of security resulting from the work of the social services and the streamlining of procedures, and of calm due to the support of the nursing assistant at times when the caregiver had to be absent. Patients experienced improved mood through the regular visits of the nurse assistant and better wellbeing because they felt cared for by the professionals. In a meta-qualitative approach involving five complex interventions with patients and caregivers, patients expressed the importance of feeling cared for as a benefit of the intervention and were grateful for and comforted by having access to healthcare services [38]. A previous study highlighted the importance of professionals being sensitive to people’s needs, empathetic and kind as important for establishing good, trusting relationships [27].

The participants in this study reported their experiences and views following the implementation of a program in a rural area of Catalonia. The lack of any participants from urban areas may have prevented other themes from being identified during the discussions. In our study, professionals realized that caregivers and patients did not associate the care received with a new model of care. This result was confirmed with the users’ interviews as we obtained opinions about the normal care provided by social and health services, which indicates that more emphasis on the program should be given to obtain more valuable information for the evaluation of the implementation process. Another limitation was the difficulty in interviewing the patients participating in the program since most of them were very old people with health problems, which made it difficult for them to take part. 

The next steps are to redesign and validate the integrated care model Salut+Social on a larger scale through a robust trial, including cost-effectiveness analyses of the intervention, and to test the model further in other areas from Spain.

## 5. Conclusions

The present study shows the acceptability by health professionals and social workers of the new Salut+Social integrated care program developed in Catalonia, as well as users’ satisfaction with the care provided. The program provided improved coordination between services by promoting regular face-to-face multidisciplinary meetings and better communication, with these activities being supported by a new app. The participants reported a reduction in unnecessary visits and in the duplication of tasks, as well as the promotion of joint home visits with better provision of services and patient care. Professionals pointed out the need for a protocol for social emergencies or hospital admission criteria and for stronger community orientation with proactive actions. Users proposed more frequent home visits.

## Figures and Tables

**Table 1:** Characteristics of the professional participants in the focus group.

Code	Gender	Age	Profession	Years Employed
P1	Female	45	Nurse	20
P2	Female	59	Nurse	26
P3	Female	46	General practitioner	20
P4	Male	42	Nurse	14
P5	Male	50	Nurse	26
P6	Female	49	General practitioner	14
P7	Female	53	General practitioner	23
P8	Female	33	Nurse	10
P9	Female	50	Social worker	20
P10	Female	43	Social worker	17
P11	Female	45	Social worker	19

P: professional.

**Table 2:** Characteristics of users (caregivers and patients) interviewed.

Code	Gender	Age	Employment	Relationship to Patient	Health Condition	Social Condition *	Home Care Program
Pt1	Female	56	Disability pensioner	-	Physical disability due to accident	Dependence degree 2	Yes
C1	Male	61	Disability pensioner	Son	Complex chronic patient, pluripathology	Dependence degree 2	Yes
C2	Female	56	Unemployed	Daughter	Complex chronic patient, pluripathology	Dependence degree 3	Yes
C3	Male	72	Retirement pensioner	Husband	Lewy body dementia	Dependence degree 3	Yes
C4	Female	54	Unemployed	Daughter	Alzheimer’s disease	Dependence degree 3	Yes
C5	Female	70	Retirement pensioner	Woman	Alzheimer’s disease	Dependence degree 3	Yes
C6	Female	42	Employed	Daughter	Complex chronic patient, pluripathology	Dependence degree 3	Yes
C7	Female	54	Unemployed	Daughter	Aneurysm, complex chronic patient	Dependence degree 2	Yes

* inclusion criterion, with degree obtained during the program; Pt: patient; C: caregiver.

## Data Availability

Not applicable.

## Supplementary Materials

The following supporting information can be downloaded at: https://www.mdpi.com/article/10.3390/ijerph192315467/s1, Supplementary File S1: Standards for Reporting Qualitative Research (SRQR). Reference [39] is cited in the supplementary materials.
